# HABs *Karenia brevis* and *Pseudo-nitzschia* pre- and post-Hurricane Michael

**DOI:** 10.2166/wh.2023.302

**Published:** 2023-04

**Authors:** Josh Alarcon, Lauren Ward, Ke Pan, Elaina Gonsoroski, Christopher K. Uejio, Leslie Beitsch, Maureen Y. Lichtveld, Emily W. Harville, Samendra Sherchan

**Affiliations:** a Department of Environmental Health Sciences, School of Public Health and Tropical Medicine, Tulane University, New Orleans, LA 70112, USA; b Department of Epidemiology, School of Public Health and Tropical Medicine, Tulane University, New Orleans, LA 70112, USA; c Department of Geography, College of Social Sciences and Public Policy, Florida State University, Tallahassee, FL 32306, USA; d Department of Behavioral Sciences and Social Medicine, College of Medicine, Florida State University, Tallahassee, FL 32306, USA; e Department of Environmental and Occupational Health, School of Public Health, University of Pittsburgh, Pittsburgh, PA 15261, USA; f Center for Climate Change and Health, Morgan State University Baltimore MD 21251

**Keywords:** disaster, harmful algal blooms, hurricane

## Abstract

Increased occurrences of harmful algal blooms (HAB) in the Gulf of Mexico, and even worldwide, yield concern for increases in brevetoxin exposure leading to respiratory illness or even death, highlighting the need for extensive scientific research and human health monitoring. It is known that major events such as tropical storms and hurricanes are followed by periods of increased red tides caused by HABs; however, the nature by which phytoplankton blooms proliferate following major events remains a topic of great interest and research. The impact of Hurricane Michael on October 10, 2018 on HABs in the Florida panhandle was examined by analyzing data from the Florida Fish and Wildlife Conservation Commission in coordination with Normalized Fluorescence Line Height (nFLH) data from the University of South Florida College of Marine Science. Results presented here demonstrate four phases of HABs during storm events: 1. Pre-storm concentrations, 2. Decreased concentration during the storm, 3. Elevated concentrations following the storm and 4. Recovery period. This time frame can serve to be important in understanding the health dynamics of coastal systems following major storm events.

## GRAPHICAL ABSTRACT

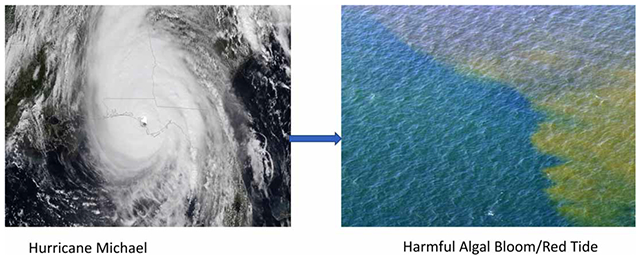

## 1. INTRODUCTION

### 1.1. Harmful algal blooms and health impacts

While algal blooms are a natural phenomenon, harmful algal blooms (HABs, ‘red tide’) occur when there is an overgrowth of species of algae that produce potent neurotoxins which can be aerosolized (inhaled) or are ingested via seafood (Weisberg et al. 2019). HABs can cause harm to marine organisms and alter food webs by the production of toxins that, once consumed by humans, can cause illness or even death (Anderson et al. 2002). Florida red tides have been documented since the 1800s; however, coastal areas in Florida have been increasingly threatened by ‘red tide’ from HABs in recent years (Fleming et al. 2011).

The frequency and magnitude of these harmful algal blooms have increased in coastal regions worldwide over the last few decades, a trend referred to as the global expansion of HABs (Anderson et al. 2002). One of the many concerns is the potential relationship between increased HABs and eutrophication of coastal waters causing increases in nitrogen (N) and phosphorus (P) as nutrient-loading fuels for high biomass algal blooms (Anderson et al. 2002). As urban and agricultural runoff continues to supply water systems with nutrients, the frequency of HAB events will continue to increase, causing significant impacts on water quality, ecosystems and human health.

*Karenia brevis*, the primary algal organism responsible for recent red tides in Florida, releases brevetoxin, a known human neurotoxin and respiratory and digestive irritant that also poisons sea life including birds, fishes and shellfish (Carvalho et al. 2010). Consumption of contaminated shellfish can cause neurotoxic shellfish poisoning (NSP), a severe acute illness which is usually self-limiting (Fleming et al. 2011). Brevetoxin also raises treatment costs for drinking water. Brevetoxin from HABs can cause mass mortalities in a variety of aquatic organisms and alter ecosystem function.

*Pseudo-nitzschia* is another algal organism responsible for the number of illnesses detrimental to sea life and human health. There are now four species of *Pseudo-nitzschia* (*P. pseudodelicatissima, P. cuspidate, P. calliantha and P. caciantha*) and many of these species produce domoic acid (DA), a neurotoxin responsible for amnesic shellfish poisoning (ASP) in humans through contaminated fish and shellfish (Dortch et al. 1997; Badylak et al. 2006; Shuler et al. 2012). Phytoplankton communities like *P. nitzschia* are also responsible for hypoxic zones. *P. nitzschia* can be found in salinities ranging from 5 to 37 ppt and temperatures between 15 and 30 °C (the smallest temperature range of the region) (Shuler et al. 2012). Similar to *K. brevis, P. nitzschia* abundance is often associated with high-nutrient input from either peak discharge or upwelling events.

Typically, tropical cyclone storms increase the abundance or distribution of HABs (Phlips et al. 2020). According to Phlips et al. (2020), high rainfall and winds associated with storms can impact a wide range of processes relevant to phytoplankton dynamics, including increased watershed runoff which increases nutrient loads. Periods of high rainfall and wind can also contain high HAB biomass from freshwater ecosystems, or conversely, increased flushing rates can reduce water residence times. However, wind damage to ecosystems can also contribute to internal nutrient loading (Phlips et al. 2020). Indeed, the monitoring of water quality, when accompanied by appropriate management actions, can assure the mitigation of ongoing HABs and the reduction of negative impacts. Little information is available on the impact of Hurricane Michael on HAB proliferation. The goal of this study is to address the impact on the abundance and distribution of HABs before and after a major hurricane.

### 1.2. Study area and Hurricane Michael

**Figure 1 |:**
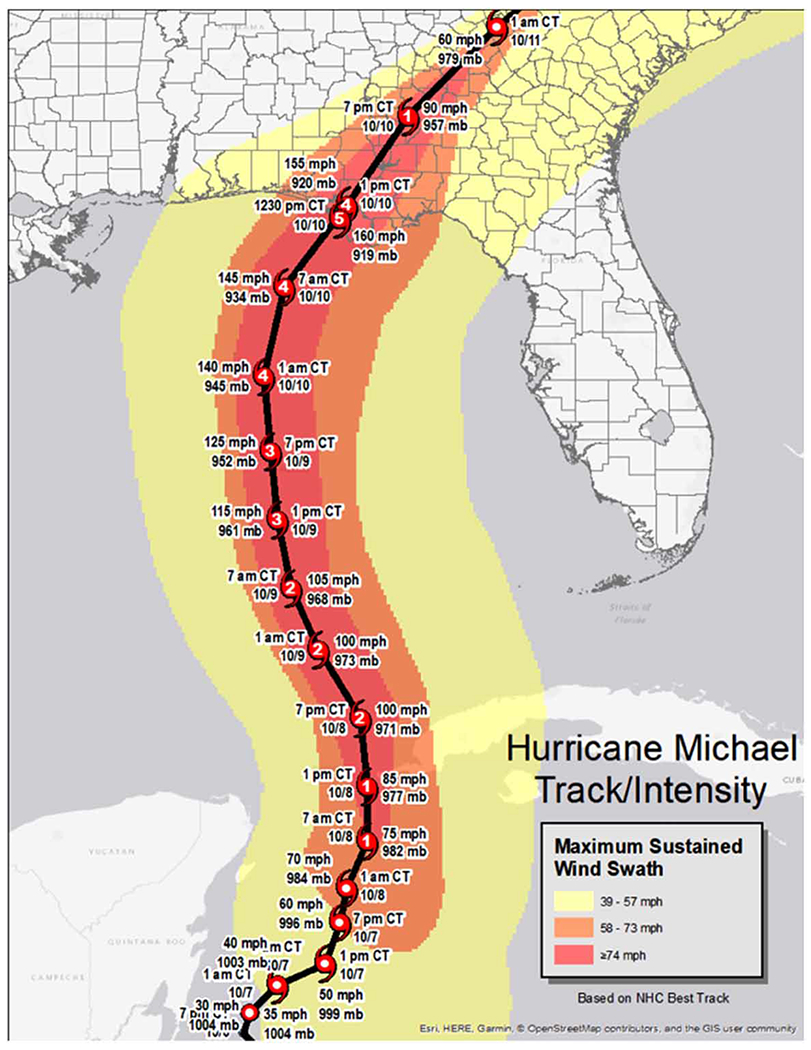
*Source*: https://www.weather.gov/tae/HurricaneMichael2018.

The Florida panhandle has, for many years, been subject to numerous hurricanes and tropical cyclones. According to Senkbeil et al. (2020), the recurrence interval of hurricane Category 3 or greater on the Saffir Simpson Hurricane Wind Scale (SSHWS) in the northwest Florida Panhandle is 21 years, an interval that is likely to decrease with ocean warming. Hurricane Michael made landfall on the Florida Panhandle on October 10, 2018, causing widespread property destruction, power outages, and storm surge-induced coastal erosion. The storm had rapidly intensified, increasing from a Category 2 to a powerful Category 5 storm in 24 h, which limited evacuation and increased the size of the population at risk (*National Weather Service*, Figure 1).

## 2. METHODS

A robust dataset collected by the Florida Fish and Wildlife Conservation Commission (FWC) beginning in 2018–2019 provided information on *K. brevis* and *P. nitzschia* along the Florida coastal counties. Data from the FFWCC was analyzed using ArcGIS and compared to real-time Normalized Fluorescence Line Height (nFLH) data provided by the University of South Florida College of Marine Science. Results were analyzed from an overall approach taking into account all 35 coastal counties in Florida, as well as a narrowed approach focusing on the four counties closest to the landfall of Hurricane Michael – Bay County, Gulf County, Okaloosa County and Walton County.

### 2.1. Fish and wildlife data

The FWC has compiled and shared a long-term database of local HAB observations. Each observation reports the sample’s geographic location in latitude and longitude, cell counts (cells/L) of *K. brevis* and other HAB species, as well as water quality characteristics. Approximately 100–150 sites measured algal concentrations every week. During a harmful algal bloom, sampling becomes much more frequent and geographically extensive, such as during the *K. brevis* bloom that initiated in November 2017 impacting Florida’s northwest, southwest and east coasts, which endured for over a year in some areas. More than 14,000 samples have been processed from the bloom’s start to the present time, providing a robust dataset for the present focus on *K. brevis* and brevetoxins since Hurricane Michael. Pre-storm samples used in this research were collected between June 2018 and September 2018, while post-storm samples were collected between October 2018 and June 2019.

### 2.2. ArcGIS

**Figure 2 |:**
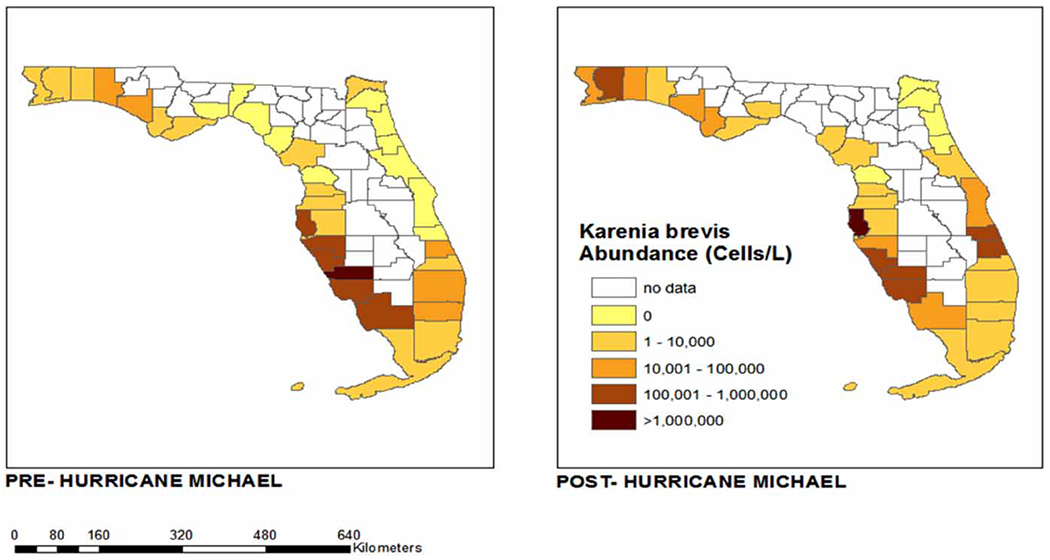
Average abundance of *K. brevis* by county in Florida pre- and post-Hurricane Michael (June 2018–June 2019). ArcGIS map of *K. brevis* abundance in coastal Florida counties before and after Hurricane Michael.

**Figure 3 |:**
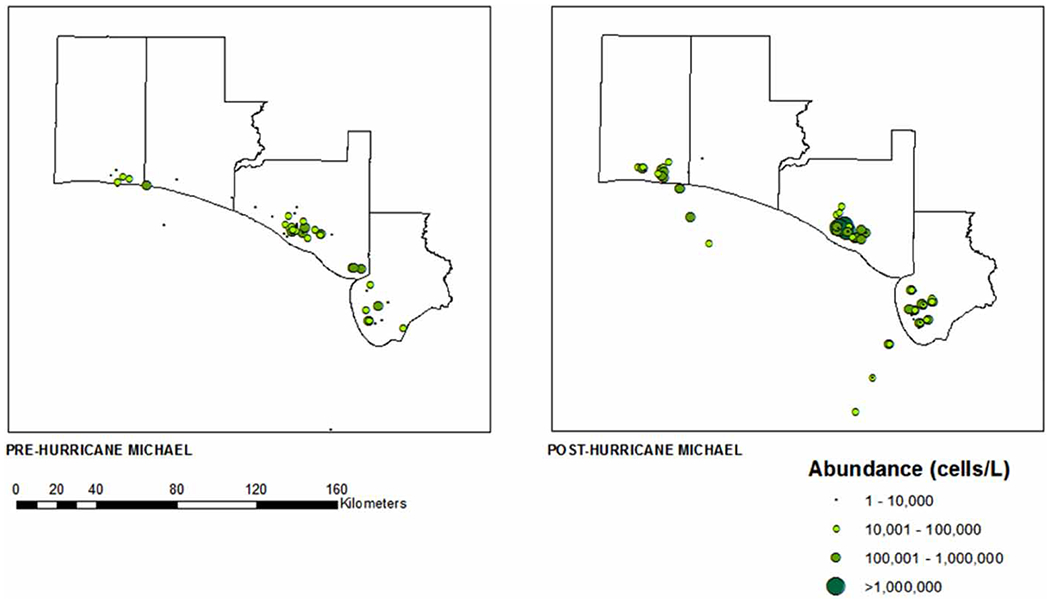
Abundance of *K. brevis* pre- and post-Hurricane Michael: Bay, Gulf, Okaloosa, and Walton counties, Florida (June 2018–June 2019). Site-level abundance of *K. brevis* in a subset of four coastal Florida counties before and after Hurricane Michael.

**Figure 4 |:**
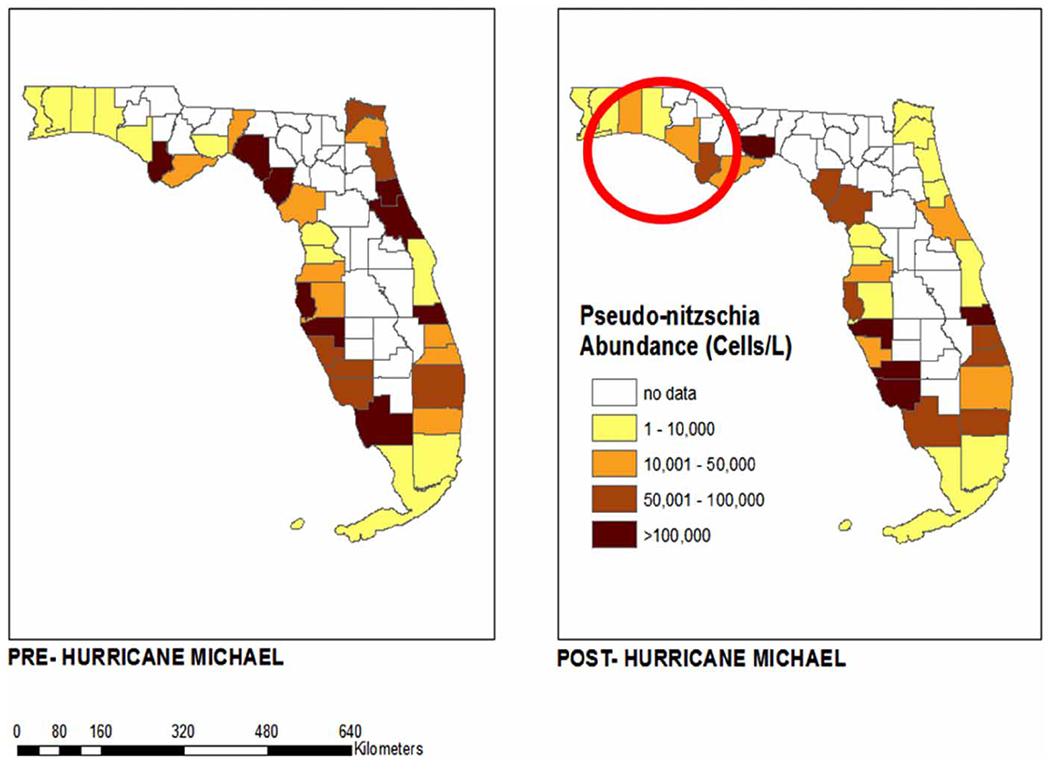
Average abundance of *P. nitzschia* by county in Florida pre- and post-Hurricane Michael (June 2018–June 2019). County-level ArcGIS map of *P. nitzschia* abundance in coastal Florida counties before and after Hurricane Michael.

**Figure 5 |:**
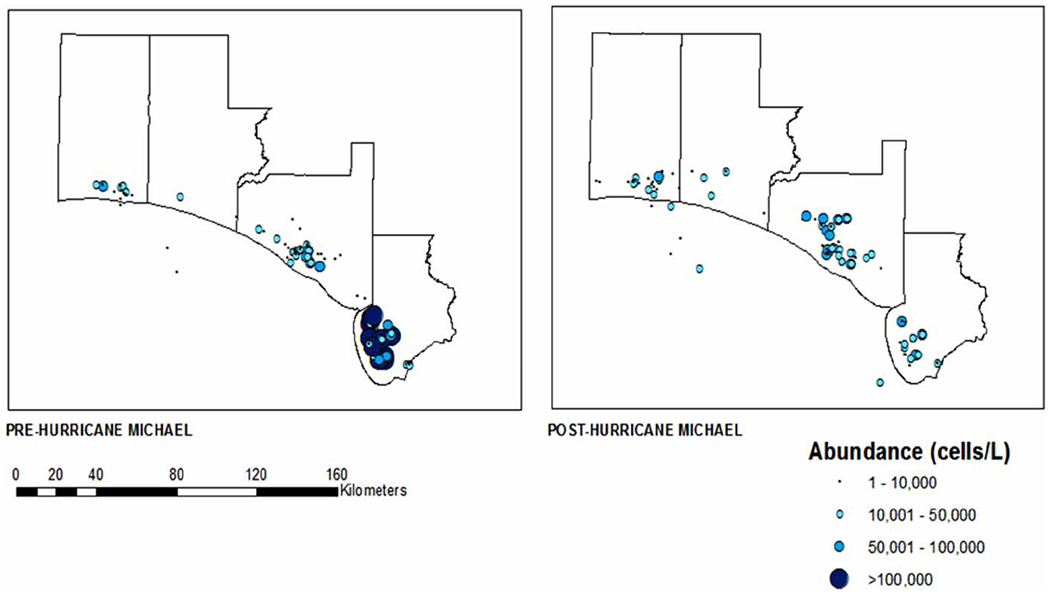
Abundance of *P. nitzschia* spp. pre- and post-Hurricane Michael: Bay, Gulf, Okaloosa, and Walton counties, Florida (June 2018–June 2019). Site-level abundance of *P. nitzschia* in a subset of four coastal Florida counties before and after Hurricane Michael.

Data points pre- and post-Hurricane Michael are plotted in ArcGIS using spatial distribution modules in order to provide a clear summary of pre- and post-storm HAB abundance and distribution of both *K. brevis and P. nitzschia*. Using geospatial data obtained from publicly available census tracts from the United States Census Bureau (https://catalog.data.gov/dataset/tiger-line-shapefile-2019-state-florida-current-census-tract-state-based), HAB observation data points were converted to .csv files and mapped onto the geographic boundaries of counties in Florida using ArcMap 10.8. The mean abundance (cells/L) of each algal organism was determined across all sites in each county. Counties bordered by the Gulf of Mexico and Atlantic Ocean statewide were mapped with a color gradient corresponding to the increasing abundance of each species (Figures 2 and 4). A sub-map of the four counties closest to the landfall of Hurricane Michael was created from the same census tract data. The abundance of both algal organisms was mapped onto the sample sites using a pattern of symbols increasing in size with increasing abundance (Figures 3 and 5).

### 2.3. Normalized Fluorescence Line Height

Analysis of this study can be verified by examining the nFLH, a standard dataset from the SeaDAS processing software provided by the University of South Florida College of Marine Science. The nFLH is a measure of solar-stimulated chlorophyll-a fluorescence, and the NASA-standard nFLH is partially corrected to account for the effect of sediment contamination. The use of chlorophyll-a as an indicator of phytoplankton abundance and eutrophication is widely used in estuarine aquatic systems as increases in chlorophyll parallel increases in nutrient concentrations (Anderson et al. 2002; Huang et al. 2011).

Any statistical methods?

## 3. RESULTS

### 3.1. Karenia brevis

In our four counties of interest, a total of 544 samples were collected before Hurricane Michael; 244 from Bay County, 208 from Gulf County, 70 from Okaloosa County and 22 from Walton County. Following Hurricane Michael, a total of 634 samples were collected; 246 from Bay County, 214 from Gulf County, 108 from Okaloosa County and 66 from Walton County. The average abundance of *K. brevis*, as illustrated in Figure 2, suggests a major redistribution of HAB abundance following Hurricane Michael. Based on the summary of cells/L, an overall increase in *K. brevis* abundance following Hurricane Michael can be seen in Figure 2. In fact, 11 of the 35 coastal counties in Florida saw an overall increase in *K. brevis* abundance following Hurricane Michael (Figure 2). Three of the four counties in the panhandle closest in proximity to the location of Hurricane Michael’s landfall (Bay, Okaloosa and Walton), as shown in Figure 3, experienced a rise in *K. brevis* abundance following the event. The abundance for Bay County increased from between 10,000 and 100,000 cells/L (median 55,000) pre-Hurricane Michael to between 10,000 and 1,000,000 cells/L (median 505,000) post-Hurricane Michael. The abundance for Okaloosa County increased from between 10,000 and 1,000,000 cells/L (median 505,000) pre-Michael to between 10,000 and >1,000,000 cells/L post-Michael. While the abundance in Walton County remained the same between 10,000 and 1,000,000 cells/L (median 505,000), the number of sites sampled (distribution) increased post-Michael (Figure 3).

### 3.2. Pseudo-nitzschia

The average abundance of *P. nitzschia*, as illustrated in Figure 4, suggests a similar trend to that of *K. brevis*. Following Hurricane Michael, *P. nitzschia* abundance increased in 8 of the 35 coastal counties in Florida, while the remaining counties showed considerable redistribution of abundance (Figure 4). Comparable to *K. brevis*, three of the four counties closest to landfall (Bay, Gulf and Okaloosa), as shown in Figure 5, experienced a rise in *P. nitzschia* abundance following the events of Hurricane Michael. The abundance for Bay, Gulf and Okaloosa counties remained the same from between 10,000 and 100,000 cells/L (median 55,000), 10,000 and 50,000 cells/L (median 30,000), and 10,000 and 100,000 cells/L (median 55,000), respectively, but the number of sites sampled (distribution) increased post-Michael. However, data suggest a dramatic decrease in abundance in Walton County from between 10,000 and >1,000,000 cells/L to between 10,000 and 100,000 cells/L (median 55,000) (Figure 7).

### 3.3. Normalized Fluorescence Line Height

**Figure 6 |:**
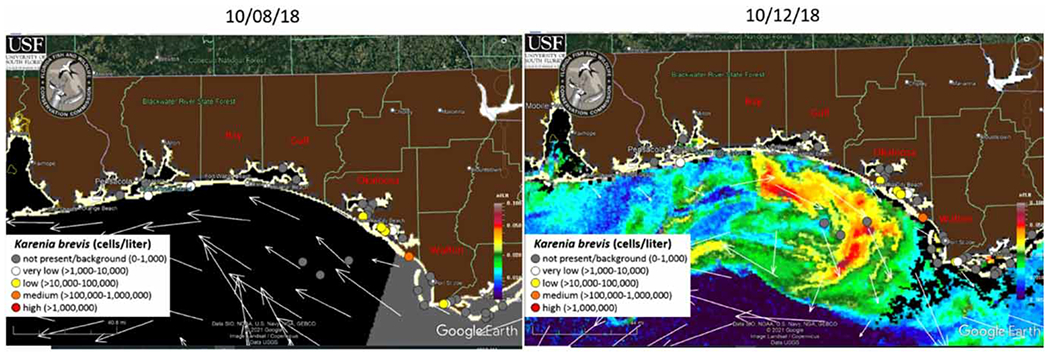
Four counties: Bay, Gulf, Okaloosa, and Walton. Pre- and post-Hurricane Michael. *Source*: https://optics.marine.usf.edu/cgi-bin/optics_data?roi=GCOOS&current=1.

Based on Figure 6, the abundance of *K. brevis* remains fairly consistent from October 8, 2018 to October 12, 2018 for the four counties in our focus, such as Bay, Gulf, Okaloosa and Walton. In fact, the abundance of *K. brevis* in Bay, Gulf and Okaloosa counties was all very low (>1,000–10,000 cells/L) to not present at all, whereas the abundance in Walton County was low (>10,000–100,000 cells/L) to medium (>100,000–1,000,000 cells/L).

**Figure 7 |:**
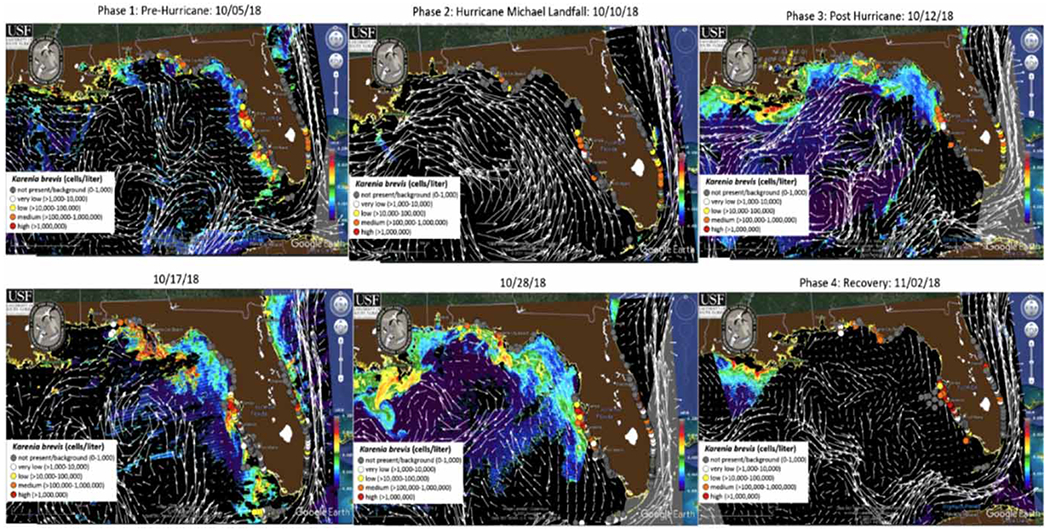
nFLH in the Gulf of Mexico during Hurricane Michael. *Source*: https://optics.marine.usf.edu/cgi-bin/optics_data?roi=GCOOS&current=1.

Even though these abundances remain the same during this time period, upon observation of the nFLH, which can also be used to determine HAB abundance, there is a massive increase in the mean chlorophyll-a concentration following Hurricane Michael on October 12, 2018. The trend shows that before the storm on October 5, 2018, the mean chlorophyll-a concentration for the open Gulf of Mexico was between 0 and 0.005 mW cm^−2^ μm^−1^ sr^−1^ and between 0 and 0.030 mW cm^−2^ μm^−1^ sr^−1^ along the four Florida coastal counties of interest (Figure 7). During the landfall of the storm on October 10, 2018, the concentration showed background levels or was undetectable along the coast. However, a substantial increase in concentrations occurs just 2 days after landfall on October 12, 2018 when mean chlorophyll-a concentration for the open Gulf of Mexico was between 0.005 and 0.010 mW cm^−2^ μm^−1^ sr^−1^ and the mean concentration along the four coastal counties of interest increased to between 0.010 and >0.050 mW cm^−2^ μm^−1^ sr^−1^(Figure 7). On 10/11/18, a change in wind direction following the storm causes a re-entry of HAB causing algae which can be observed by the nFLH explosion on October 12, 2018. This occurrence is not only observed in the four counties of interest but also along the entire Gulf of Mexico North American coastline from the tip of Florida to the southern portions of Texas (Figure 7).

## 4. DISCUSSION

The focus of this research was to monitor and analyze the proliferation of HABs in comparison to nFLH data during a major storm event. Because the chlorophyll-a fluorescence measured by the nFLH stimulates the growth of HABs, the nFLH is often used to infer HAB abundance. During Hurricane Michael, both HABs and nFLH are not present or have background (0–1,000 cells/L) or very low (>1,000–10,000 cells/L) concentrations due to unfavorable conditions such as temperature, wind and waves. Following the storm, a return of nFLH levels to the Gulf Coast 2 days after the passing of Hurricane Michael allowed for the growth and return of HABs. From October 12 to October 28, the expansion of HABs along the coast becomes more widespread and an increase in *K. brevis* cases medium (>100,000–1,000,000 cells/L) to high (>1,000,000 cells/L) concentrations with the highest observed concentrations on the central west coast (Figure 7) is observed. This corresponds to the increase in chlorophyll-a values from mostly ~0.010 mW cm^−2^ μm^−1^ sr^−1^ with small areas as high as 0.050 mW cm^−2^ μm^−1^ sr^−1^ on October 10, 2018 to between 0.010 and 0.040 mW cm^−2^ μm^−1^ sr^−1^ with greater occurrences of concentrations of 0.050 mW cm^−2^ μm^−1^ sr^−1^.

When comparing this post-hurricane data to a pre-hurricane algal bloom on October 5, conclusions can be made that the levels or concentrations of HABs following the Hurricane event are much higher than levels before the storm. It is not until November 2, 23 days after the storm, that the levels begin to drop off again (Figure 7). This information is useful in regard to making inferences and correlating the strength and magnitude of hurricanes such as Hurricane Michael to the duration of an elevated HAB period following hurricanes and tropical/extratropical storms. For this particular storm, it was nearly a month until the Florida coast began to see ‘normalized’ or pre-storm levels of HABs.

While the number of samples observed in this study is greater post-Hurricane Michael, the trends observed using ArcGIS are validated upon the observation of the nFLH data. With the collaboration of this data, it is possible to speculate that there are four phases involving HABs during a storm event: 1. Pre or ‘normal’ concentrations, 2. Decreased concentration during the storm, 3. Elevated concentrations following the storm and 4. Recovery period. During the third phase of this process, as mentioned previously, three out of the four counties of interest, as well as the majority of Florida’s 35 coastal counties, showed an increase in *K. brevis* and *P. nitzschia* concentrations.

This data can be corroborated by Huang et al. (2011) who found similar trends with Hurricane Ivan in Pensacola in 2004. Huang et al. (2011) found that mean chlorophyll-a levels were three to four times higher (mean 14.7 μg/L and as high as 20 μg/L) after the passing of Hurricane Ivan than levels before the storm (5.3 μg/L). Huang et al. (2011) go on to note that hurricanes can cause heavy stormwater runoff, which carries nutrient loads into the bay, and storm surge inundation which together simulates a bloom in chlorophyll-a.

While the majority of HABs initiate mid-continental shelf (Weisberg et al. 2019), coastal topography, including estuaries, rivers and coastal lakes, impacts the distribution of phytoplankton plumes, especially in places like Florida that have numerous rivers, estuaries, bays, and coastal lakes. River diversion can particularly increase nutrient loading, having major impacts on phytoplankton biomass (Bargu et al. 2019); however, diatom species will not dominate for long if there is a mixing of freshwater and saltwater (Bargu et al. 2011) or vertical mixing of the water column (Lewis et al. 1984). Periods of lower rainfall with higher salinities and water residence times provide favorable conditions for phytoplankton blooms, while periods of high rainfall or watershed discharge can restrict phytoplankton biomass due to unfavorable conditions (Phlips et al. 2020). This is consistent with the trend observed here, as chlorophyll-a and phytoplankton concentrations were the below detection limit during the storm and followed by a period of elevated concentrations. In fact, the *K. brevis* bloom along the western coast of Florida in 2018 was considered one of the worst red tide events since 2005 (Weisberg et al. 2019).

## 5. CONCLUSION

The number of samples collected and analyzed after Hurricane Michael suggests that three out of the four (3/4) counties of interest showed an increase in *K. brevis* and *P. nitzschia* post-Hurricane Michael. The nFLH parallels this finding, indicating an explosive increase in chlorophyll-a following storm coinciding with a change in the wind direction during the passing of the storm. Lastly, the data presented here suggest four phases of HAB abundance during major events like hurricanes: 1. Pre or ‘normal’ abundance, 2. Decreased concentration during the storm, 3. Elevated concentrations following the storm and 4. Recovery period. Considering the increase in the frequency and magnitude of harmful algal blooms in coastal areas, which are subject to morphological change and natural weather phenomena, understanding the proliferation of HABs during such events becomes crucial in minimizing the impacts posed to environmental and human health.

## Data Availability

All relevant data are included in the paper or its Supplementary Information.

## HIGHLIGHTS

- Impact of Hurricane Michael on algal blooms in the Florida panhandle was examined.
- Four phases of HABs during storm events were found.
- This time frame can serve to be important in understanding the health dynamics of coastal systems following a hurricane.
